# The Story of the Insane from Year to Year

**Published:** 1896-05-23

**Authors:** 


### THE STORY OF THE INSANE FROM YEAR TO YEAR.

NINTH SERIES.

### THE ROYAL ASYLUM, ABERDEEN.

On January 1st there were 714 patients in this asylum, and by the end of the year the number had risen to 741, of whom 240 were private patients. There were 195 admissions, 81 recoveries, and 50 deaths. The recoveries stand at 41·5 per cent. of the admissions, and the deaths at 7 per cent. on the average number resident. Of the deaths, 20 were from cerebral diseases, 4 from consumption, and 3 from pneumonia. Hereditary influence accounts for 73 of the admissions, drink for 23, and love for 2. It is most pleasing to us to give publicity to the following statement taken from the chairman's report: "No patient belonging to the county of Aberdeen for whom the rate of £30 a year can be paid from private means is refused admission. If such patients were not received in this asylum as private patients, they would either have to be left without asylum treatment, or their relatives would be driven to make application to the parochial authorities to deal with them as persons requiring public aid, and the patients would thus have the stigma of pauperism attached to them." It is the want of such accommodation in England that so often necessitates this stigma on many people who greatly dislike it, and who would gladly pay £30 a year to avoid it. The Commissioners say, "it was everywhere evident that the institution is managed with great care and marked ability."

